# Periscope of Dermatology

**Published:** 1897-06-05

**Authors:** 


### PERISCOPE OF DERMATOLOGY.

(*Continued from pag3 148.*)

**Therapeutics.**—Gelanthum: This soluble varnish,[35] recommended by Unna, consists of equal parts of gelatine and tragacanth. Each substance is macerated slowly, then subjected to steam and filtered. By these means the gelatine is deprived of a part of its gelatinous property. The two substances are then mixed, and subjected to steam for two days, and then pressed through gauze. Glycerin 5 per cent., some rose water, and 2 per cent. of thymol are added. The medicament to be added, if not soluble in water, must be rubbed into a soft paste with water, and fats must first be emulsified by gums. The varnish spreads easier, dries quicker, and with a smoother surface than the older varnishes, and tolerates all substances singly or together.

**Picric Acid.**—Maclennan[36] has employed picric acid locally in the treatment of acute eczema, and in other superficial inflammatory affections. Owing to the powerful astringent properties which this chemical possesses, it forms a protective layer of coagulated albumen, under which healing rapidly proceeds, and its antiseptic properties prevent suppuration; it is free from danger, and causes not the slightest pain; all itching and smarting abate almost immediately.

Sack[37] recommends the following mixture as a perfect solution of coal-tar. It has a pleasant aromatic odour, anl is unirritating to the skin. ℞.: Coal tar, 10 parts; benzene, 20 parts; acetone, 77 parts ♏. Cases of intense pruritus and extensive lichenification, ringworm, disidrosis, and various seborrhœic affections are benefited by its use. Salicylic acid, with or without resorcin, can be added when there is considerable hyperkeratosis.

Gaucher[38] finds that acetone collodium is the best recipient for oil of cade; it does not stain linen, and the odour of the acetone to some extent disguises that of the oil of cade. He uses the preparation in psoriasis, lichen, and chronic eczema.

**Tumours.**—Crocker[39] describes a case of myoma multiplex of the skin, and quotes other recorded cases of the disease. He finds that seven were females and four were males, and the age at which tumours commenced to develop varied from infancy to 60 years of age. The size of the tumours varied, but there was a strong tendency to group into irregular patches or bands. The colour was usually brownish-red, and the tumours were in the skin, but freely moveable with it over subjacent parts. They seldom increase to a larger size than that of a pea, and do not return after removal. There is a strong tendency to most severe paroxysmal pain in the growths.

Shield[40] records a case of Paget's disease of the breast in a woman age 47. The malady commenced six years before, after bursting of an abscess. The patch measured 11 inches vertically and 10½ inches horizontally. The nipple was destroyed by ulceration, and there was a fungating growth at the orifice of the old sinus. Shield[41] also records a case of Paget's disease or dermatitis maligna of the skin of the abdominal wall. The patient was 60 years of age, and in feeble health. For eight years he had suffered from a sore place over the pubes, which had slowly extended till it measured five inches horizontally and three inches vertically. It extended over the root of the penis and down the scrotum. It had a red glazed surface, and on it were three somewhat granulomatous tumours. After removal the tumours were found to be epitheliomatous, and the skin presented the appearance of Paget's disease on microscopic examination.

Besnier,[42] in recording a case of mycosis fungoides, calls attention to the following practical diagnostic points: (1) In all cases of doubtful itching skin disease not amenable to ordinary treatment, whether like eczema, psoriasis, lichenoid prurigo, or urticaria, the question of the possibility of mycosis fungoides in an early stage must be considered. (2) The initial premycosic stage is not an incubation, but is the disease in action. In the case recorded tumours did not appear till 16 or 17 years after the onset of the itchy and cutaneous eruptions. Death took place a year later.

**Various.**—Freeman[43] describes a dermatitis due to the handling of hyacinth bulbs. It consists in a marked erythema, which appears within five minutes of contact, and sometimes goes on to the formation of wheals. The cause of the eruption is a parasite, about the size of the acarus scabei, which swarms in the dust about the bulbs.

Shield[44] describes the presence of erythema on the arms after they have been speckled with arterial blood. He frequently has observed it on his own person, and it appears after the blood has been washed away. It is not attended by itching, burning, or inconvenience.

Harrison[45] also reports the occurrence of blood erythema in a surgeon after sprinkling with arterial blood. After washing away the blood a pink stain was left, which lasted for 15 minutes or longer. There was also some irritation. The same surgeon was also very susceptible to the smell of blood, and could detect the occurrence of hæmorrhage in a house by the smell. Only arterial blood produced the condition.

Ohmann-Dumesnil[46] describes some extreme cases of cornu cutaneum and of cornu unguale. He points out that marked cases are now rare, as they are treated early. With regard to treatment, they should be freely excised, and the base treated by means of an electric or Paquelin's cautery. In the case of cornu unguale, the whole of the matrix of the nail should be thoroughly destroyed by the cautery.

[35] Brit. Med. Journ., Oct. 17, 1896; Int. Med. Mag., Oct., 1896. [36] Brit. Med. Journ., Dec. 26, 1896; Ditto, March 6, 1897. [37] Med. Week, Oct. 30, 1896. [38] Brit. Med. Journ. Ep., Jan. 9, 1897. [39] Brit. Journ. of Derm., Jan., 1897, p. 1, and Feb., p. 48. [40] Ditto, Nov., 1896, p. 443. [41] Ditto, Jan., 1897, p. 35. [42] Amer. Journ. of Med. Sci., Feb., 1897, p. 232. [43] Brit. Journ. of Derm., Feb. 1897, p. 66. [44] Ditto, Nov. 1896, p. 430. [45] Ditto, Jan., 1897, p. 42. [46] Tri-State Med. Journ., Nov., 1896, p. 519.

